# Computational analysis of perturbations in the post-fusion Dengue virus envelope protein highlights known epitopes and conserved residues in the Zika virus

**DOI:** 10.12688/f1000research.8853.2

**Published:** 2016-09-14

**Authors:** Sandeep Chakraborty

**Affiliations:** 1 Celia Engineers, Navi Mumbai, India

**Keywords:** Zika, Dengue, flaviviruses, microcephaly, Guillain-Barre Syndrome, envelope proteins, epitopes, neutralizing antibodies

## Abstract

The dramatic transformation of the Zika virus (ZIKV) from a relatively unknown virus to a pathogen generating global-wide panic has exposed the dearth of detailed knowledge about this virus. Decades of research in the related Dengue virus (DENV), finally culminating in a vaccine registered for use in endemic regions (CYD-TDV) in three countries, provides key insights in developing strategies for tackling ZIKV, which has caused global panic to microcephaly and Guillain-Barre Syndrome. Dengue virus (DENV), a member of the family *Flaviviridae*, the causal agent of the self-limiting Dengue fever and the potentially fatal hemorrhagic fever/dengue shock syndrome, has been a scourge in tropical countries for many centuries. The recently solved structure of mature ZIKV (PDB ID:5IRE) has provided key insights into the structure of the envelope (E) and membrane (M) proteins, the primary target of neutralizing antibodies. The previously established MEPP methodology compares two conformations of the same protein and identifies residues with significant spatial and electrostatic perturbations. In the current work, MEPP analyzed the pre-and post-fusion DENV type 2 envelope (E) protein, and identified several known epitopes (His317, Tyr299, Glu26, Arg188, etc.) (MEPPitope). These residues are overwhelmingly conserved in ZIKV and all DENV serotypes, and also enumerates residue pairs that undergo significant polarity reversal. Characterization of α-helices in E-proteins show that α1 is not conserved in the sequence space of ZIKV and DENV. Furthermore, perturbation of α1 in the post-fusion DENV structure includes a known epitope Asp215, a residue absent in the pre-fusion α1. A cationic β-sheet in the GAG-binding domain that is stereochemically equivalent in ZIKV and all DENV serotypes is also highlighted due to a residue pair (Arg286-Arg288) that has a significant electrostatic polarity reversal upon fusion. Finally, two highly conserved residues (Thr32 and Thr40), with little emphasis in existing literature, are found to have significant electrostatic perturbation. Thus, a combination of different computational methods enable the rapid and rational detection of critical residues as epitopes in the search for an elusive therapy or vaccine that neutralizes multiple members of the *Flaviviridae* family. These secondary structures are conserved in the related Dengue virus (DENV), and possibly rationalize isolation techniques particle adsorption on magnetic beads coated with anionic polymers and anionic antiviral agents (viprolaxikine) for DENV. These amphipathic α-helices could enable design of molecules for inhibiting α-helix mediated protein-protein interactions. Finally, comparison of these secondary structures in proteins from related families illuminate subtle changes in the proteins that might render them ineffective to previously successful drugs and vaccines, which are difficult to identify by a simple sequence or structural alignment. Finally, conflicting results about residues that are involved in neutralizing a DENV-E protein by the potent antibody 5J7 (PDB ID:3J6U) are reported.

## Introduction

The genus Flavivirus of the family *Flaviviridae* comprises of more than 70 viruses, including important human pathogens such as the Zika (ZIKV), Dengue (DENV), Japanese encephalitis (JEV), yellow fever (YFV), Tick-borne encephalitis (TBEV) and West Nile (WNV) viruses ^1, 2^. Currently, only four flaviviruses (YFV, TBEV, JEV and DENV) have licensed vaccines ^3, 4^. In flaviviruses, a single polyprotein encoded by a positive-sense RNA genome is cleaved by viral and host proteases into three structural (premembrane:prM, envelope:E and core:C) and seven non-structural (NS1, NS2A, NS2B, NS3, NS4A, NS4B, and NS5) proteins ^5^. These Class II fusion viruses ^6^ enter the cell through clathrin-mediated endocytosis ^7, 8^, triggered by protonation of conserved histidine residues at low pH ^9, 10^. Conformational changes of E-homodimers to E-monomers at the viral surface expose a highly conserved fusion loop ^11^, which subsequently penetrates the outer leaflet of the host membrane ^12^, wherein a stable trimer creates a fusion pore allowing the nucleocapsid to enter the cytosol ^13^. Subsequent to viral replication, virus assembly creates nonfusogenic immature particles in the lumen of the endoplasmic reticulum. The host protease furin in the trans-Golgi network converts this non-virulent form to a smooth virulent virion by cleaving the globular prM into pr and M proteins, of which the M protein remains associated with meta-stable E homodimers ^5, 14^.

Until recently, ZIKV infections were rare and confined to Asia and Africa ^15^. An analysis of the 2007 ZIKV outbreak in Yap Island, Federated States of Micronesia concluded with the prophetic warning that ‘clinicians and public health officials should be aware of the risk of further expansion of Zika virus transmission’ ^16^. The dramatic transformation of this relatively unknown virus to a globally recognized pathogen occurred after it was detected in Brazil ^17^, and quickly spread across the globe (Brazil, France, United States of America, and El Salvador to- date), prompting a WHO emergency committee to assess the linkage of this virus to microcephaly and Guillain-Barré syndrome (GBS) ^18, 19^.

This sudden crisis has exposed the dearth of detailed knowledge about ZIKV. Computational homology modeling has been used to address this limitation exploiting the large volume of data available on related viral structures ^20^. While the genome of ZIKV was sequenced in 2007 ^21^, the structure of mature ZIKV ^22^ was only recently determined, elucidating several salient features of the E and M proteins, the target of most neutralizing antibodies ^23– 25^. However, decades of research on other members of the Flavivirus family provides a trove of information that needs to be contextualized with respect to ZIKV.

DENV has four serotypes (DENV1-4) ^26^. The essential challenge in developing a tetravalent DENV vaccine has been the fact that antibodies for a particular serotype can be enhancing, and potentially life-threatening for secondary infections with other serotypes ^27^. Apart from vaccines, other anti-viral strategies include developing peptide vaccines ^28^, using peptide-inhibitors derived from the viral proteins ^29^, inhibiting the fusion process ^30^ and anionic peptides that target cationic ‘hotspots’ ^31, 32^. Computational epitope predictors like the sequence based RANKpep ^33^ or the structure based Pepitope ^34^ have been used to validate antibody binding ^35, 36^. A detailed structural analysis of proteins of these flaviviruses will provide deeper insight into conservation than a sequential analysis does. Furthermore, analyzing the spatial and electrostatic perturbations of protein structures after conformational changes arising due to the fusion process helps in identifying residues that are critical and possibly exposed to the environment, making them better candidates as vaccine epitopes.

In the current work, several computational methods were used to analyze DENV and ZIKV E protein structures. Firstly, a quantitative analysis of spatial and electrostatic perturbation in the pre ^37^ and post-fusion ^12^ DENV-2 E proteins was done using MEPP ^38^. This revealed that highly perturbed residues are overwhelmingly conserved, and also epitopes of known neutralizing antibodies ^23, 35, 39– 43^. Characterization of *α*-helices in E-proteins using techniques (PAGAL ^44, 45^) previously applied to the Ebola virus ^46^) revealed that *α*1 in ZIKV-E and DENV-E proteins is not conserved in the sequence space. Furthermore, *α*1 is perturbed in the post-fusion protein in DENV2-E protein ^12^, and includes a known epitope that is not part of the pre-fusion *α*1 ^41, 42, 47^. PAGAL analysis also highlights a cationic *β*-sheet within a putative GAG-binding domain ^48, 49^, which consists of a pair of arginine residues that have significant electrostatic polarity reversal ^48, 49^. Finally, residues that are involved in antibody neutralizing by 5J7 were re-analyzed, and some conflicting results were obtained ^50^.

## Methods

The MEPP (version 1) ^38^ and PAGAL (version 1) ^44^ packages have been previously described. The recently solved cryo-EM structure of ZIKV (PDB ID:5IRE) was used as the main structure for analysis of ZIKV in the current study ^22^. PDB ID:1OKEA was the structure of the DENV2-E protein used for analyzing domains I-III, which lacks the stem and transmembrane domains ^37^. The structure of post-fusion DENV2-E protein was obtained from PDB ID:1OK8A ^12^. Since the post-fusion DENV2-E protein did not have side-chains densities for residues 145-158, these residues were removed from the pre-fusion protein (PDB ID:1OKEA) in order to have an uniform comparison (see 1OKEAFIXED.pdb in Dataset1). This has the implicit assumption that this loop effects both pre-fusion and post-fusion proteins in the same manner. A radius of 6Å was used to identify interacting residues ^38^. The ‘distance perturbation index’ is computed by dividing the absolute distance deviation with the smaller of the distances.

For the stem and transmembrane domains, a DENV3 (PDB ID:3J6SA) structure was used. Since PDB ID:3J6SA has resolution of 6Å and no side-chain atoms, SWISS-MODEL ^51^ was used to generate the model of PDB ID:3J6SA using the ZIKV-E protein (PDB ID:5IREA) as the template (see 3J6SASWISSA.pdb in Dataset1). Hardware requirements are very modest - all results here are from a simple workstation (8GB ram) and runtimes were a few minutes at the most.

The APBS (v1.4) ^52, 53^ parameters were set as described previously in 54. APBS writes out the electrostatic potential in dimensionless units of kT/e where k is Boltzmann’s constant, T is the temperature in K and e is the charge of an electron. All protein structures were rendered by PyMOL(TM) Molecular Graphics System, Version 1.7.0.0. ( http://www.pymol.org/). *α*-helices and *β*-sheets were extracted using DSSP (version 2.2.1) ^55^. Protein structures have been superimposed using MUSTANG (mustang-3.2.1) ^56^. The color coding for the Edmundson wheel is as follows: all hydrophobic residues are colored red, while hydrophilic residues are colored in blue: dark blue for positively charged residues, medium blue for negatively charged residues and light blue for amides.

Multiple sequence alignment was done using MAFFT (v7.123b) ^57^, and figures generated using the ENDscript server ^58^. In order to obtain a multiple sequence alignment with a single representative of a stereochemical group (positive, negative, aromatic and non-polar residues) the following substitutions were done: E *>*D, R *>*K, S *>*T, W *>*F, Y *>*F, L *>*M, V *>*M, I *>*M, A *>*M. Gly (without a side chain) and Pro (with a cyclic side chain) were not substituted. His was also not substituted, due to its importance in pH sensing among flaviviruses ^9, 10^. PHYML (v3.0) was used to generate phylogenetic trees from alignments ^59^.

## Results

Raw data for ‘MEPPitope: spatial, electrostatic and secondary structure perturbations in the post-fusion Dengue virus envelope protein highlights known epitopes and conserved residues in the Zika virus’ README.txt contains a description of the files. Click here for additional data file. Copyright: © 2016 Chakraborty S 2016 Data associated with the article are available under the terms of the Creative Commons Zero "No rights reserved" data waiver (CC0 1.0 Public domain dedication).

**Figure 1.**
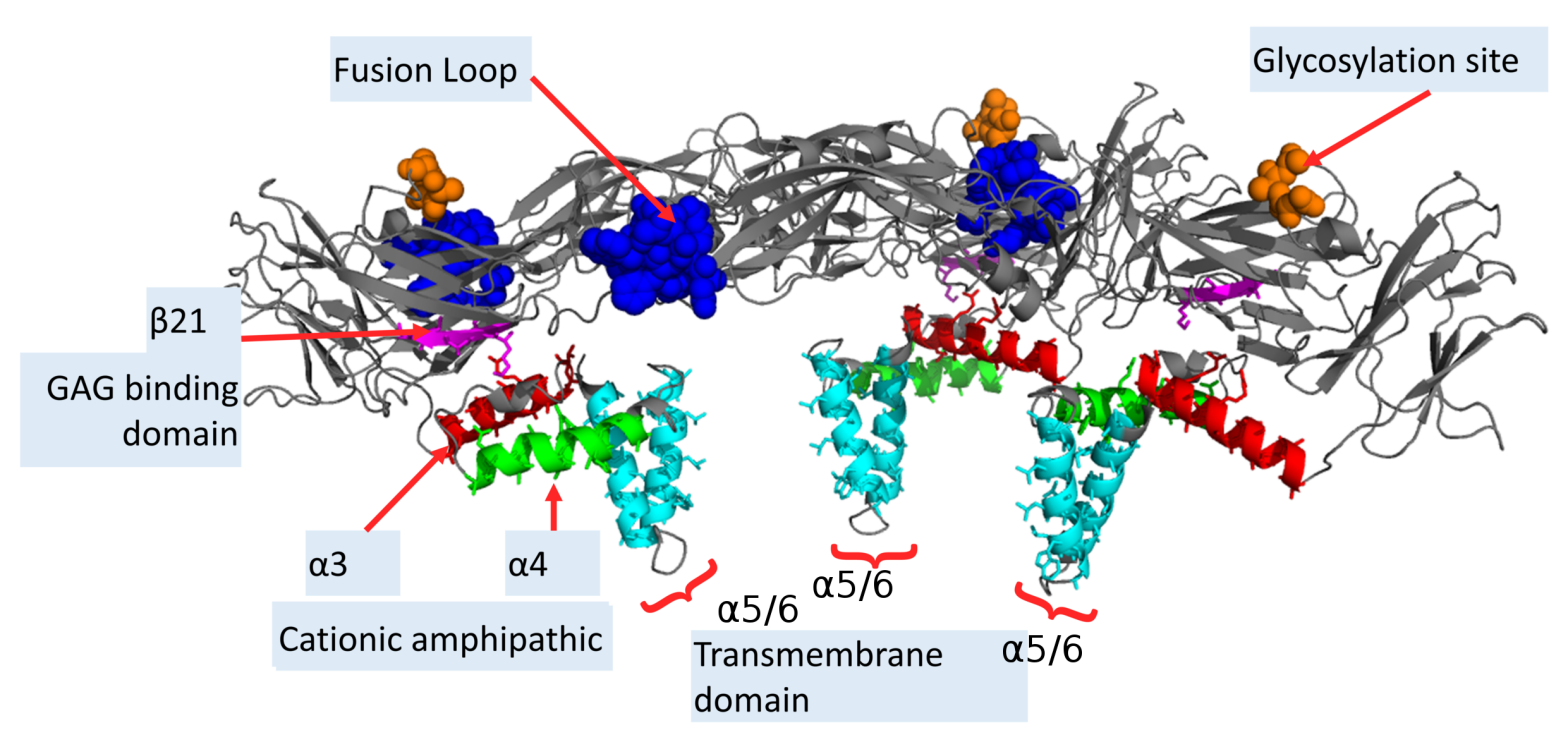
The structure of the ZIKA-E protein (PDB ID:5IRE). The E-proteins form a raft-like structure, in complex with the M-proteins (not shown here). Most common exposed residues are the highly conserved fusion loop (residues 98-109 in blue), the glycosylation site (Asn154 in ZIKV, in orange), and the GAG-binding domain which consists of a cationic *β*-sheet (in magenta). The stem region consists of cationic amphipathic helices *α*3 and *α*4 in the E proteins in red and green, respectively. The hydrophobic transmembrane helices *α*5 and *α*6 are in cyan.

**Figure 2.**
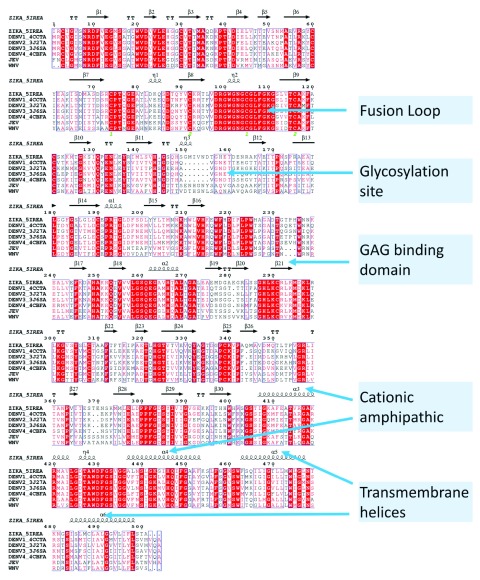
Multiple sequence alignment (MSA) of envelope (E) proteins from ZIKA/DENV1-4/JEV/WNV. The most prominent difference between the E protein from DENV and other viruses analyzed here is a missing stretch of amino acids near the Asn153 glycosylation site. This stretch is the possible reason for an incorrect alignment of the conserved glycosylation site (N-x-S/T) sequence in the MSA (both ClustalW and MAFFT has this issue). Also, DENV has an additional glycosylation site (Asn67) missing in other viruses. MSA was done using MAFFT ^57^, and the alignment of the secondary structures were done using ESPript ^58^.

The focus of the study in the current paper is the ZIKV and DENV envelope (E) protein, a determinant of tropism and virulence ^60^. Unless explicitly specified, residue numbering is based on DENV2 (PDB ID:1OKEA), while secondary structures are numbered according to the ZIKV protein (PDB ID:5IREA). Each E-protein subunit is about 500 residues long in these flaviviruses. The soluble ectodomain has three distinct domains (I, II, III) - domain I and II are interlaced in the sequence space ^61^. These domains are followed by a stem region which contains two cationic amphipathic helices separated by a stretch of conserved sequences ^62, 63^, ending in an anchor region with two transmembrane helices ( Figure 1, Figure 2). Apart from a conserved glycosylation site (Asn153) present in all flaviviruses, DENV has an additional site for N-linked glycosylation (Asn67) which regulates interaction with the lectin DC-SIGN ^64^. The hydrophobic anchoring fusion loop (residues 98-109), which penetrates the outer bilayer leaflet of the host cell membrane to initiate cell entry ^65^, is highly conserved in all flaviviruses ( Figure 1, Figure 2).

### Analysis of spatial and electrostatic perturbation in the post fusion DENV-E protein

**Figure 3.**
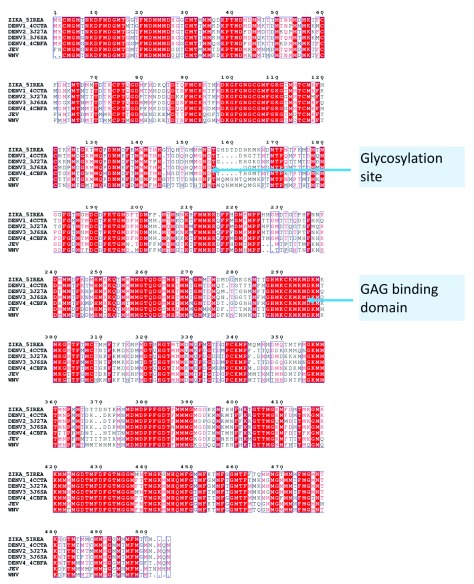
Multiple sequence alignment (MSA) of envelope (E) proteins from ZIKA/DENV1-4/JEV/WNV after substituting for stereochemical equivalence. The following substitutions were done in the sequence space: E>D, R>K, S>T, W>F, Y>F, L>M, V>M, I>M, A>M in order to use a single amino acid for positive, negative, aromatic and non-polar residues. Gly (without a side chain) and Pro (with a cyclic side chain) were not substituted. His was also not substituted, due to its importance in pH sensing among flaviviruses ^9, 10^. These substitutions enable MAFFT to align the glycosylation site properly. Also, these show the stereochemical equivalence of the cationic residues *β*-21 in ZIKV (PDB ID:5IREA), which is part of the GAG-binding domain.

The pre-(PDB ID:1OKE ^37^) and post-(PDB ID:1OK8 ^12^) fusion conformations of DENV2 were used for MEPP analysis ^38^. The major difference in these proteins is a 33Å displacement of domain III, as previously noted ^9, 13^. Several metrics were used for identifying residues that undergo spatial, electrostatic and secondary structure perturbations. The first analysis computed pairs of residues that have a electrostatic potential difference (EPD) reversal (EPD-R) (> 150 units), were within 8 Å of each other in both conformations and had minimal distance perturbation (<4Å). Residues were marked as (i) completely conserved, (ii) stereochemically equivalent or (iii) not conversed. His317, the residue implicated in pH sensing ^66, 67^, switches electrostatic polarity with respect to Thr315 ( Table 1). Both His317 and Thr315 are conserved in ZIKV/DENV1-4/JEV/WNV ( Figure 2), and are known epitopes ^23, 43^. Another pair (Arg286-Arg288) with EPD-R are stereochemically equivalent in ZIKV/DENV1-4/JEV/WNV ( Figure 3), and lie on a putative GAG-binding domain preceding the DI/DIII linker ^48, 49^. Thr359, which is an epitope for the same MAb that binds Thr315 and His317 ^23^, but is not conserved even among DENV serotypes, is another such residue which has EPD-R with Ser363 ( Figure 2). Thus, barring the pair Thr32-Thr40 ( Table 1), all residues that have an EPD-R with respect to a spatially proximal residue are known to be epitopes, even when not conserved across different viruses.

**Table 1.** Residue pairs with reversal in electrostatic potential difference (EPD) in the post-fusion DENV2 E-protein. These pairs have minimal distance perturbation (<4 Å), significant reversal in EPD (> 150 units) and are within 8 Å in both conformations. For example, Arg286-Arg288, part of a cationic *β*-sheet and a putative GAG-binding domain, has an electrostatic perturbation without having any relative spatial displacement. F-: final value in post-fusion DENV-E protein (PDB ID:1OK8A), O-: original value in pre-fusion DENV-E protein (PDB ID:1OKEA). Conserved in ZIKV and all four DENV serotypes? - Y: yes, N: no, StCh: stereochemically equivalent. Distances in Å. See Methods section for units of potential.

PDB ID:1OK8A	PDB ID:1OKEA	F-EPD	O-EPD	*δ*EPD	F-DIST	O-DIST	*δ*-DIST	Conserved
ARG/286/NH1 THR/315/OG1 THR/40/OG1	ARG/288/NH1 HIS/317/ND1 THR/32/OG1	96.1 164.6 151.8	-68.5 -70.6 -134.7	164.6 235.2 286.5	5.6 4.1 5.3	5.7 4.6 7.7	-0.1 -0.5 -2.4	StCh:StCh Y-Y Y-Y
THR/359/OG1	SER/363/OG	104.6	-56.0	160.6	5.1	7.0	-1.9	N-N

**Figure 4.**
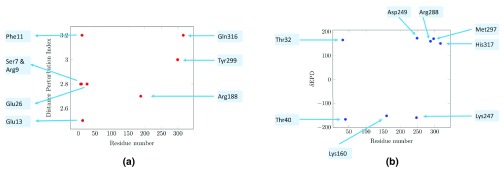
Spatial and electrostatic perturbations in the post-fusion DENV2-E protein analyzed using MEPP. MEPP analyzes the spatial and electrostatic potential difference (EPD) deviations of each residue with respect to other residues in close proximity (< 6Å). ( **a**) Distance deviation. Barring Ser7, all residues are completely conserved in ZIKV and DENV. All residues, barring Gln316, are known epitopes. Gln316 precedes the histidine residue responsible for initiating pH driven conformational changes during cell entry. ( **b**) Electrostatic perturbation. Several residues identified by electrostatic features do not have known references as epitopes in current literature. Thr32 and Thr40 are two such residues, which are conserved in these flaviviruses.

Next, normalized distance deviations (see Methods) highlight Phe11, Tyr299, Ser7, Arg9, Glu26, Arg188, Glu13 and Gln316 as residues with the largest spatial perturbations ( Figure 4a). Barring Ser7, all residues are completely conserved in ZIKV/DENV1-4 ( Figure 2). A N-terminal peptide (DENV3,4-12 VGVGNRDFV) that enhances immunogenicity for CD8+ T cells when expressed from modified vaccinia Ankara includes Phe11, Ser7 and Arg9 ^39^. Arg9 and Glu13 are also epitopes of other antibodies ^35^. This particular study also showed that the N8R substitution DNA vaccine had a more neutralizing and protective effect than wild-type immunized sera, both *in vitro* and *in vivo* ^35^. Arg9 is part of a salt bridge with Glu368 which maintains the structure of the E-protein in the pre-fusion state ^10, 66^. Tyr299 is part of the epitope for the cross-reactive neutralizing MAb DENV1-E102 ^68^. Arg188 is essential for infectivity, and is neutralized by DC4 Fab ^40^, while the monoclonal antibody DD18-5 recognized residue Glu26 in DENV4 ^35^, a residue predicted by the Pepitope server ^34^. Thus, all spatially perturbed residues identified by MEPP are known epitopes.

The following residues have significant cumulative EPD deviations ( *>*150 EPD units) with other residues within 6Å - Thr32, Thr40, Lys160, Lys247, Asp249, Arg288, Met297 and His317 ( Figure 4b). His317, Arg288, Thr32 and Thr40 have been discussed above. Several residues identified by this electrostatic feature do not have known references in current literature. Of these residues, Lys160 and Met297 are not conserved in ZIKV/DENV1-4, while Asp249 is conserved in DENV, but not in ZIKV ( Figure 2). However, Thr32 and Thr40 are two conserved residues ( Figure 2) with EPD deviations, leading to an EPD-R as described above ( Table 1).

**Figure 5.**
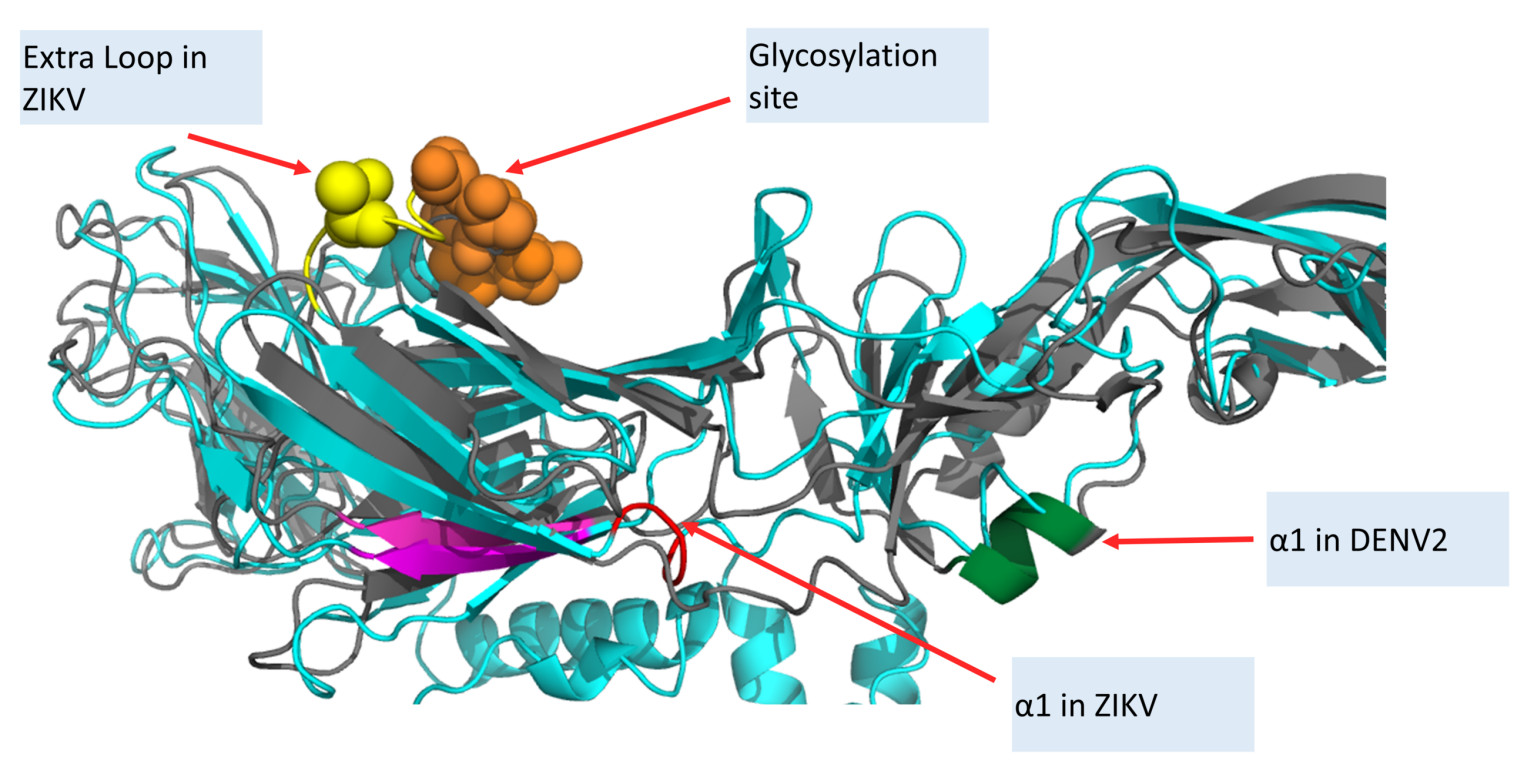
Major structural differences between ZIKV and DENV E-proteins. ZIKV (PDB ID:5IREA) in cyan, DENV2 (PDB ID:1OKEA) in magenta are superimposed using MUSTANG ^56^. An extra loop is present in ZIKV/JEV/WNV ( Figure 3), and absent in DENV, near the glycosylation site. *α*1 in ZIKV (residues 192-195) and *α*1 in DENV2 (residues 210-214) are not conserved. α1 of DENV2 increases in length by one post-fusion, and includes the known epitope Asp215.

Subsequently, analysis of *α*-helices in the pre- and post-fusion DENV2-E protein revealed *α*1 is slightly perturbed post fusion, increasing in length by one residue (Asp215) compared to the pre-fusion *α*1 ( Figure 5, Table 2). Asp215 is important for infectivity ^42^, a proven ^41^ and predicted ^47^ epitope, and a membranotropic region of the E protein (peptide 29) ^69^.

**Table 2.** Features of *α*-helices in envelope (E) proteins from DENV2 and ZIKV. The soluble ectodomain has two *α*-helices - *α*1 and *α*2. *α*1 is perturbed in the post-fusion DENV2, increasing in length by one to include the known epitope Asp215. Moreover, *α*1 is not conserved in the sequence space of ZIKV-E. *α*2 remains conserved all E-proteins, even after fusion. *α*3/ *α*4 are amphipathic and cationic. The transmembrane helices ( *α*5/ *α*6) with no charged residues have a low hydrophobic moment. HM: Hydrophobic moment, RPNR: Relative proportion of positive residues among charged residues, Len: length of the *α*-helix, NCH: number of charged residues.

PDB ID	Feature	AH	Start	End	Len	HM	RPNR	NCH
1OK8A (DENV2:post-fusion)	Asp215+	*α*1 *α*2	210 257	215 263	6 7	2.2 2.1	0.5 0.5	2 2
1OKEA (DENV2:pre-fusion)	Asp215-	*α*1 *α*2	210 257	214 263	5 7	1.9 2.1	1 0.5	1 2
5IREA (ZIKV:pre-fusion)	Diff from DENV2 amphipathic cationic amphipathic cationic uncharged hydrophobic uncharged hydrophobic	*α*1 *α*2 *α*3 *α*4 *α*5 *α*6	192 262 407 437 463 484	195 268 423 453 477 498	4 7 17 17 15 15	2.8 2.1 10.4 6 2.1 1.3	1 0.5 0.8 1 -1 -1	1 2 5 2 0 0

### Analysis of secondary structures

**Figure 6.**
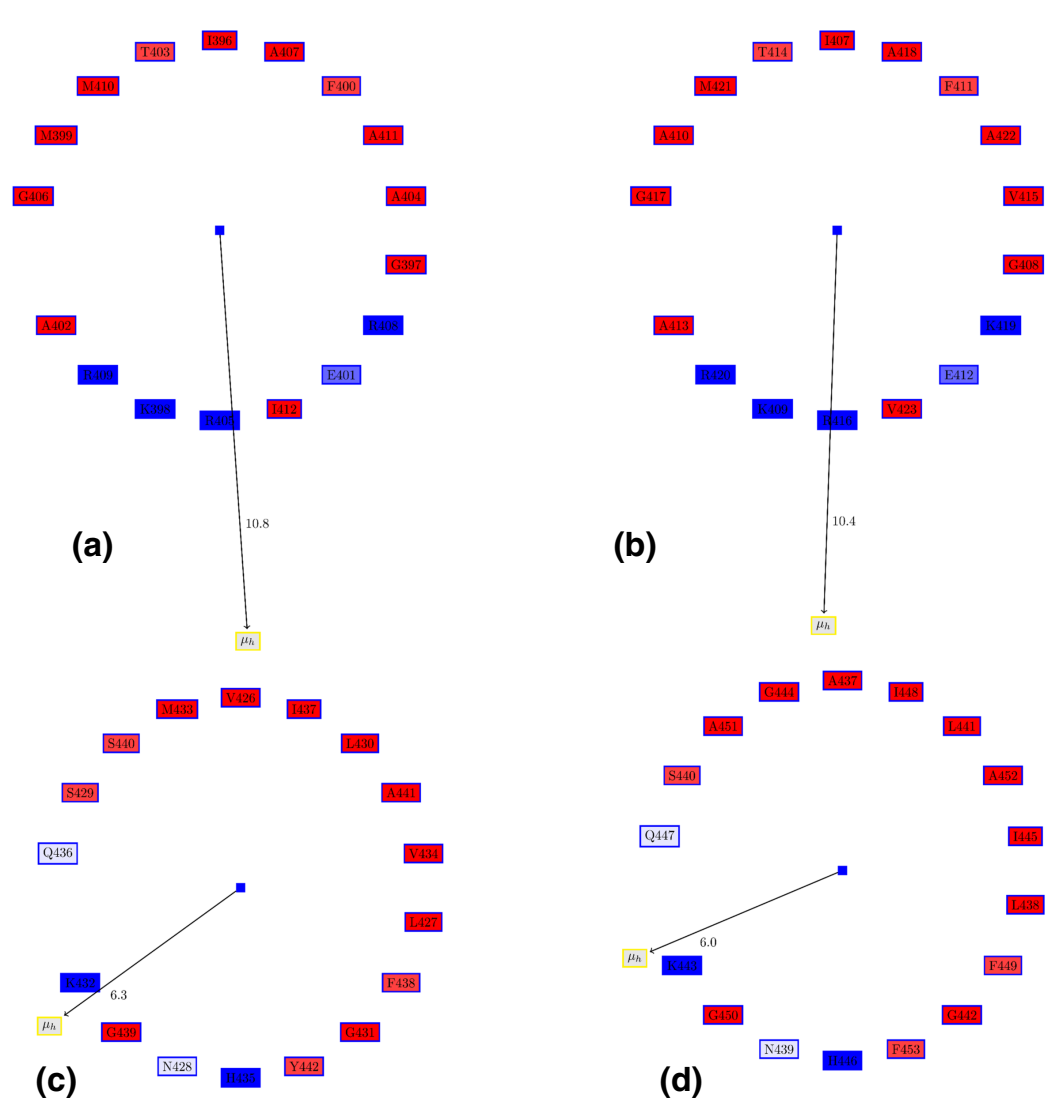
Edmundson wheel of the cationic amphipathic *α*-helices in the stem region of ZIKV-E (PDB ID:5IREA) and DENV3-E (PDB ID:3J6SA) proteins. ( **a**) *α*3 in DENV-E protein. ( **b**) *α*3 in ZIKV-E protein. ( **c**) *α*4 in DENV-E protein. ( **d**) *α*4 in ZIKV-E protein. The Edmundson wheel shows the amphipathic cationic nature of the stem helices. The hydrophobicity of residues in the hydrophobic face is an important determinant of virulence ^63, 72, 73^. The conservation of the charged face of *α*4 ( **c** and **d**) is in contrast to several differences in the hydrophobic face. The color coding for the Edmundson wheel is as follows: all hydrophobic residues are colored red, while hydrophilic residues are colored in blue: dark blue for positively charged residues, medium blue for negatively charged residues and light blue for amides.

The secondary structures from DENV and ZIKV E-proteins were extracted using DSSP ^55^, and analyzed using PAGAL ^44^. The ZIKV-E protein (PDB ID:5IREA, length=501 residues) has six *α*-helices and thirty *β*-sheets (see SSEinfo.zip in Dataset 1). The Edmundson wheels ^70^ for these *α*-helices in the stem region ( Table 2) shows their amphipathic cationic nature ( Figure 6). Interfacial hydrophobicity plays a critical role in cell entry of viruses ^71^. The membranotropic *α*3 and *α*4 in DENV4 ^69^ has been studied extensively through mutational studies of the hydrophobic face ^62, 63^. Another strategy using peptide mimetic (residues 412 to 444, named DN59) derived from these helices showed inhibition of flaviviruses by releasing genomic RNA ^72, 73^. A similar study based on peptide mimetic of residues 419-447 (comprising the conserved stretch following *α*3 and *α*4) inhibited viral entry ^74^. These peptides were most effective at inhibition when three residues (442-444) were mutated to tryptophan, the most hydrophobic residue according to the Wimley-White whole residue hydrophobicity scale ^75^. An interesting feature of *α*4 is the complete conservation of residues on the charged surface - Ser439, Gly436, Lys432, Gly439 and H435 in DENV ( Figure 6), while the hydrophobic face is much more variable. Only Asn428 is not conserved ( Figure 2).

**Figure 7.**
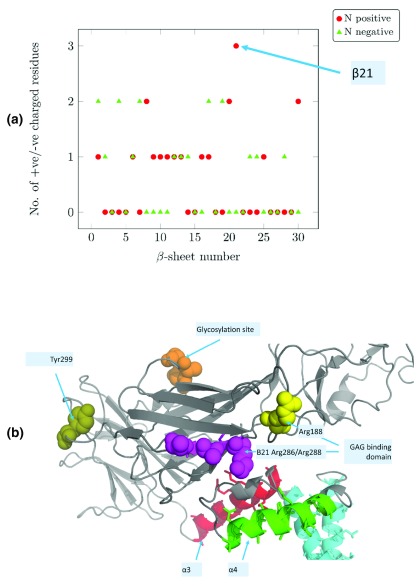
Charged profile of *β*-sheets in the ZIKV-E protein (PDB ID:5IREA). ( **a**) *β*-21 in ZIKV-E is the most distinctive, and has three positively charged residues (290:KCRLK). The stereochemical nature of these residues are conserved in ZIKV/DENV1-4/JEV/WNV ( Figure 3). ( **b**) *β*21 is part of the GAG-binding domain that precedes the domain I/III linker, and is physically proximal to the cationic *α*4. This sheet has a pair (DENV:Arg286-Arg288) with electrostatic polarity reversal post-fusion. A mutation of Tyr299 to Phe299 disrupted virus-like particle assembly, although it was compensated by a mutation in *α*3 (DENV-E Q400H), which is distant from Tyr299.

ZIKV and DENV are Class II fusion viruses that deploy *β*-sheet-rich domains to destabilize membranes ^6^. The charged features of these *β*-sheets emphasizes *β*21 in ZIKV (294:KCRLK, preceding domain I/III linker) as distinctive, since it has three positively charged residues ( Figure 7a). Two arginine residues on this putative GAG-binding domain ^48, 49^, stereochemically equivalent in ZIKV/DENV1-4/JEV/WNV ( Figure 3), was identified by MEPP as having a significant electrostatic polarity reversal after membrane fusion ( Table 1). This residue pair (Arg286–Arg288) remains on the *β*-sheet post-fusion. This cationic ‘hotspot’ might be the target of small anti-viral anionic peptides ^31, 32^. A separate study focused on mutations in the DI/DIII linker demonstrated that a compensatory mutation in *α*3 (DENV-E Q400H) restored virus-like particle assembly disrupted by a mutation (DENV-E Y299F). Interestingly, DENV-E Q400 is not conversed even among DENV serotypes ^76^, and Tyr299 is distant from *α*3 ( Figure 7b).

### Comparing E proteins in flaviviruses

**Supplementary Figure 1.**
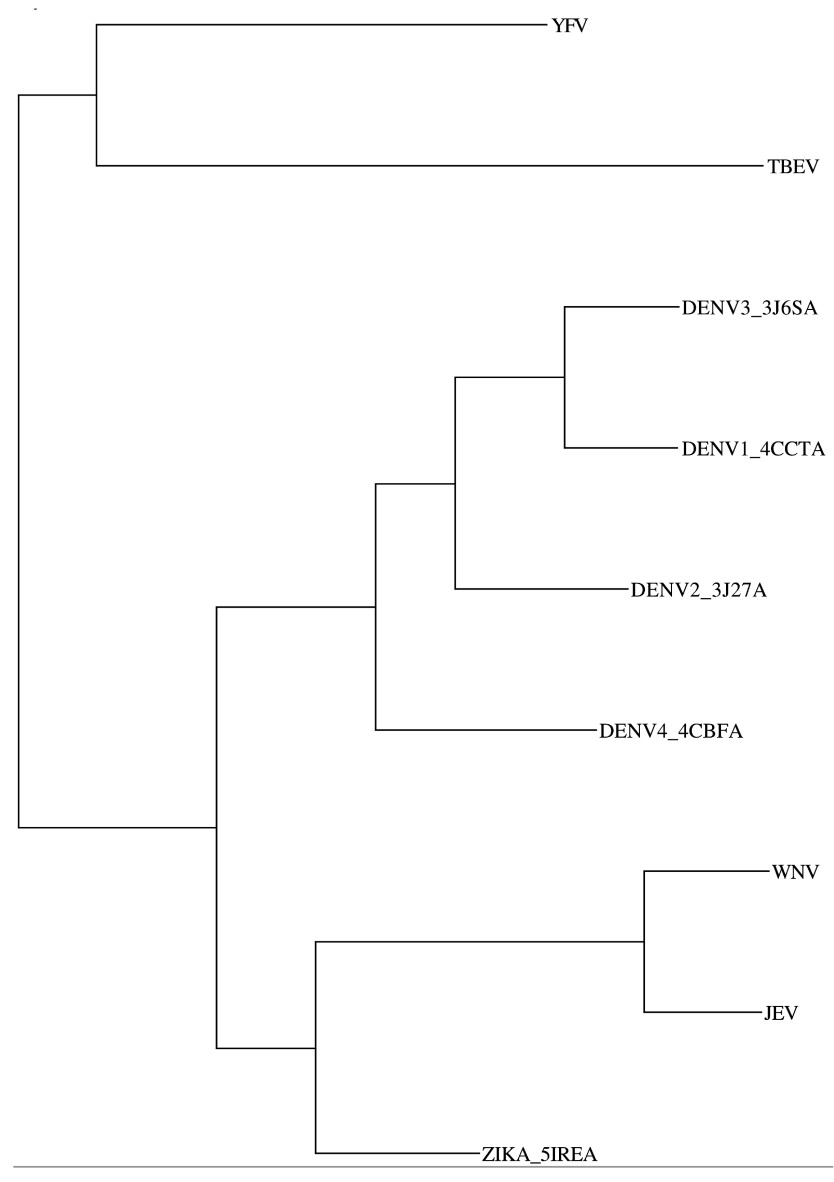
Phylogeny of different flaviviruses based on the E-protein sequence alignment. Japanese encephalitis virus (JEV, GenBank Accid:AAZ17558.1), West Nile virus (WNV, GenBank Accid:ACH99530.1), Tick-borne encephalitis virus (TBEV, GenBank Accid:AEP25269.1), Yellow fever virus (YFV, GenBank Accid:AAA92696.1). TBEV and YFV are distantly related to DENV, ZIKV, JEV and WNV.

The phylogenetic tree for these flaviviruses derived from the multiple sequence alignment (MSA) of the E-protein shows that TBEV and YFV are related, and distant from ZIKV/DENV1-4/JEV/WNV (see Supplementary material Figure 1). TBEV and YFV were excluded from the MSA. Excluding TBEV and YFV shows that ZIKV, JEV and WNV have a loop near the glycosylation site which is missing in DENV ( Figure 2). However, both ClustalW ^77^ and MAFFT ^57^) failed to align the glycosylation residues correctly. Replacing stereochemically equivalent residues (see Methods) corrected this alignment, and also gave a better visualization of conservation and differences ( Figure 3).

### Conflicting data in comparison to previous studies

**Supplementary Figure 2.**
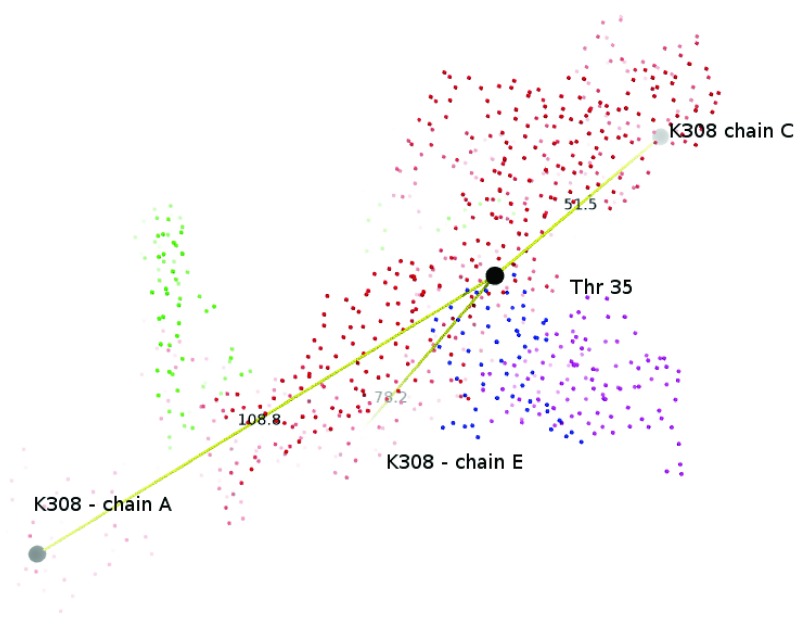
Conflicting data on interacting residues of HMAb 5J7 and E-proteins (PDB ID:3J6U). Side-chain densities were not resolved for cryo-EM Fab 5J7–DENV3 map since it had a 9 Å resolution. It is reported that ‘a slightly negatively charged patch formed by T35, S37, S82 and S84 of the heavy chain interacted with a positively charged patch formed by K307 and K308 of the E protein’, by considering interacting residues between Fab and E proteins where pairs of C *α* atoms are < 8 Å. However, the C *α* atoms of these residues are quite far. For example, T35 from 5J7 is 108, 51 and 78 Å away from K308 in the three E-proteins (PDB ID:3J6UA, 3J6UC and 3J6UE), respectively.

A recent study on the DENV3-specific human monoclonal antibody 5J7 demonstrated a very potent neutralizing effect through the binding of envelope proteins (PDB ID:3J6U) ^50^. Interacting residues were determined based on a distance of 8Å since side-chain densities were not resolved (cryo-EM Fab resolution was 9Å). Table 1 in the study reported that T35 from the heavy chain of 5J7 (PDB ID:3J6UH) interacts with four residues (Q52, Q131, E133, N134) from the DENV-E protein (PDB ID:3J6UC), and with K307 and K308 from another E-protein of the same complex. While T35 was within 10 Å for Q52, Q131, E133 and N134, the data on K307 and K308 could not be reproduced since T35 was found to be at a much larger distance from K308 in all three subunits (see Supplementary material Figure 2). The interacting residues of the heavy chain (PDB ID:3J6UH) and the light chain (PDB ID:3J6UL) with other subunits, as computed in this study (see Supplementary material Table 1 and Supplementary material Table 2, respectively). Distance-sorted interacting residues indicates Thr51 in the DENV3-E protein (chain C) is closest to the heavy chain (H-chain) ( Table 3). This explains the specificity of 5J7 to DENV3, since Thr51 is found only in DENV1 and DENV3. Another interacting residue, Thr223, is not conserved in any other DENV or ZIKV virus ( Figure 2). A different study using only the DENV3 domain III identified K307 and K308 as binding sites for mAb 14A4-8 in DENV3, but also included other domain III residues (K325, A329, G381 and I387) not present in 5J7 binding of DENV3 ^78^. It was recently shown that a domain III-specific antibody protected mice from ZIKV infection ^79^.

**Table 3.** Interacting residues of the potent DENV antibody 5J7 (PDB ID:3J6U) with other subunits, sorted based on distance. Thr51 in the DENV-3 protein (chain C) is closest to the Leu109 of the heavy chain (H-chain), and Thr223/Thr224 in the DENV-3 protein (chain C) is closest to Ile101 in the light chain (L-chain). Thr224 is conserved in ZIKV and the other DENV serotypes. However, Thr51 is conserved only in DENV1, while Thr223 is not conserved in ZIKV or other DENV serotypes, explaining the lack of neutralization of other serotypes by 5J7. The H-chain also binds to the conserved fusion loop of another DENV3-E protein (chain A).

E subunit	Antibody	E atom	Antibody atom	Distance Å
C C C C C A C A A C	H-chain H-chain L-chain H-chain H-chain H-chain H-chain H-chain H-chain L-chain	THR/51/CA THR/51/CA THR/224/CA THR/51/CA GLN/52/CA GLY/104/CA LEU/53/CA GLY/106/CA GLY/106/CA THR/223/CA	LEU/109/CA GLU/108/CA ILE/101/CA LEU/110/CA GLU/108/CA ASN/63/CA LEU/110/CA SER/37/CA VAL/61/CA TYR/100/CA	3.9 5.0 5.1 5.2 5.3 5.3 5.3 5.3 5.4 5.5

### Limitations and conclusions

Spatial congruence of catalytic residues in the active site of functionally equivalent proteins, even with no sequence homology ^80^, has been long established ^81^. Further, electrostatic potential difference (EPD) ^52, 53^ was also shown to be conserved in cognate pairs of active site residues in these active sites ^54, 82, 83^. Comparison of apo and holo structures quantifying the spatial and electrostatic perturbations after ligand binding was shown to identify critical catalytic residues in several enzymes ^38^.

In the current work, this basic postulate was extended to posit that perturbed residues in viral envelope proteins during fusion with the host membrane are good candidates as epitopes for vaccines (MEPPitope). Specifically, computational methods ^38, 44, 55^ were used to analyze spatial, electrostatic and secondary structure perturbations between a pre- ^37^. and post-fusion ^12^ DENV2-E protein. These residues are overwhelmingly conserved in ZIKV and all DENV serotypes ( Figure 2), and are known epitopes ^23, 35, 39– 43^. While perturbation was found to be a good predictor of an epitope, not all epitopes are perturbed. For example, the current study did not identify any residues in the fusion loop, the target of several neutralizing antibodies ^61, 84– 87^, or Thr51/Thr224 ( Table 3) that is an epitope of a potent neutralizing antibody ^50^. The structure of ZIKV-E protein with the ligand 2A10G6, a flavivirus broadly neutralizing murine antibody, also reveals the fusion loop as an epitope ^88^. The hydrophobic fusion loop sequence is highly conserved in all flaviviruses ( Figure 2), demonstrating the importance of sequence alignment as a strategy to identify epitopes ^89^. The current study identified few perturbed residues in domain III (only His317 and Thr315) as significantly perturbed, consistent with the observation that although antibodies targeted to domain III endow protection and minimize enhancement when present, they are redundant and can be replaced by neutralizing antibodies targeted to other epitopes on the virion ^90^. This study indicates two residues (Thr32 and Thr40) as a significantly perturbed pair in terms of its electrostatic profile. Thr32 is conserved in all flaviviruses, while Thr40 in all conserved in all except TBEV, where it is the stereochemically equivalent Ser40. There has been no emphasis on these residues as epitopes in previous literature. In summary, the current study presents a computational methodology to extract structural and electrostatic features of envelope proteins that undergo conformational changes during fusion, which correlates well with known epitopes of DENV. Conservation of such residues in ZIKV provides a good strategy to leverage existing knowledge in developing ZIKV specific therapeutics.

## Data availability

The data referenced by this article are under copyright with the following copyright statement: Copyright: © 2016 Chakraborty S

Data associated with the article are available under the terms of the Creative Commons Zero "No rights reserved" data waiver (CC0 1.0 Public domain dedication). 

*F1000Research*: Dataset 1. Raw data for ‘MEPPitope: spatial, electrostatic and secondary structure perturbations in the post-fusion Dengue virus envelope protein highlights known epitopes and conserved residues in the Zika virus’, 10.5256/f1000research.8853.d123549 ^91^

## Figures and Tables

**Supplementary Table 1.** Interacting residues of the heavy chain (PDB ID:3J6UH) of the potent DENV antibody 5J7 with other subunits. Interacting residues are identified as those with C *α* atoms within 8 Å of each other. The heavy chain binds to the A (PDB ID:3J6UA) and C (PDB ID:3J6UC) chains of the DENV-E protein, as well as the light chain (PDB ID:3J6UL).

H-chain	DENV-E Chain A	DENV-E Chain C	L-chain
T35 S37 S38 Y39 T40 Q50 G51 L52 E53 W54 G56 G57 P60 V61 F62 N63 N66 Y67 A68 Y102 D106 K107 E108 L109 L110 F111 S112 R113 A114 F115 D116 W118 G119 Q120 G121	G106 G106 G106 C74 G104 C105 G106 S72 R73 C74 G104 C105 G106 G104 C105	Q52 E133 N134 Q52 L53 A54 Q131 Q52 L53 Q52 A54 Q52 T51 Q52 L53 A54 T51 Q52 L53 E49 A50 T51 Q52 L53 G273 T274 A50 T51 Q52 L53 A54 K128 V130 T274 T198 N270 G273 T274	Q107 G106 Q107 G108 Y94 F105 G106 Q107 T104 F105 P102 Y103 T104 F105 P102 I101 P102 P102 P102 P102 A50 Y39 Y56 G57 Y39 N41 Y56 G57 S98 Q99 N41 L53 Y56 G57 S98 L53 L53 A50 P51 A50 K49 A50 K49 A50

**Supplementary Table 2.** Interacting residues of the light chain (PDB ID:3J6UL) of the potent DENV antibody 5J7 with other subunits The C *α* atoms of interacting residues are within 8 Å of each other. The C-chain of the DENV-E protein interacts with both the heavy and light chains.

L-chain	DENV-E Chain C (3J6UC)	H-chain (3J6UH)
D8 S35 S37 Y39 N41 K49 A50 P51 L53 Y56 G57 S60 Y94 S98 Q99 Y100 I101 P102 Y103 T104 F105 G106 Q107 G108	E225 E123 E123 S271 G273 T223 T224 E225 A222 T223 T224 E225 T224 E225	S112 R113 R113 A114 Q120 G121 Y102 W118 G119 Q120 G121 W118 A114 F115 D116 S112 R113 A114 S112 R113 A114 L52 R113 A114 R113 G57 W54 G56 G57 N66 Y67 A68 W54 E53 W54 L52 E53 W54 G51 L52 Q50 G51 L52 G51
