# Supplementary material for: Computational analysis of perturbations in the post-fusion Dengue virus envelope protein highlights known epitopes and conserved residues in the Zika virus
Source: F1000Res. 2016 Sep 14;5:1150. Originally published 2016 Jun 3. [Version 2] doi: 10.12688/f1000research.8853.2 (PMC4965698; doi:10.12688/f1000research.8853.2)
Supplement: Peer review history

# Peer review: Referee response for version 1

**DOI:** 10.5256/f1000research.9528.r15317

**Published:** 2016-07-29

**Authors:** Yi Shi

**Affiliations:** 1 Chinese Academy of Sciences Key Laboratory of Pathogenic Microbiology and Immunology, Institute of Microbiology, Chinese Academy of Sciences, Beijing, China

In the manuscript entitled " MEPPitope: spatial, electrostatic and secondary structure perturbations in the post-fusion Dengue virus envelope protein highlights known epitopes and conserved residues in the Zika virus", Sandeep Chakraborty analyzed the pre- and post-fusion DENV type 2 envelope (E) protein, and identified several known epitopes, which are conserved in ZIKV and all DENV serotypes. Perturbations of spatial and electrostatic or secondary structure identified by a combination of different computational methods help to detect critical residues that can be made target of therapies.

 Overall, the paper provides interesting data. The paper could be improved by addressing the following points:

1. The title is too long and needs to be shortened.
2. There are some new antibodies against ZIKV or DENV reported recently, the paper would benefit by adding new information.
3. α 5 and 6 are not labeled in Figure 1.
4. In the section of “conflicting data in comparison to previous studies” line 9, the statement “… from another E-protein of the same subunit” is inaccurate as subunit refers to a single polypeptide chain.

I have read this submission. I believe that I have an appropriate level of expertise to confirm that it is of an acceptable scientific standard.

# Response

**Published:** 2016-09-08

**Authors:** Sandeep Chakraborty

**Affiliations:** Tata Institute of Fundamental Research, India

Dear Dr Shi,

 I would like to thank you for taking the time to review this paper, and providing constructive criticism on the overall manuscript. I have revised the manuscript based on the comment of all three reviewers.

 Please find my responses below.

**In the manuscript entitled " MEPPitope: spatial, electrostatic and secondary structure perturbations in the post-fusion Dengue virus envelope protein highlights known epitopes and conserved residues in the Zika virus", Sandeep Chakraborty analyzed the pre- and post-fusion DENV type 2 envelope (E) protein, and identified several known epitopes, which are conserved in ZIKV and all DENV serotypes. Perturbations of spatial and electrostatic or secondary structure identified by a combination of different computational methods help to detect critical residues that can be made target of therapies.**

****

**Overall, the paper provides interesting data. The paper could be improved by addressing the following points:**

****

**The title is too long and needs to be shortened.**

     >> Done.

       

**There are some new antibodies against ZIKV or DENV reported recently, the paper would benefit by adding new information.**

 >> Ref 79 and 88 have been added.

****

**α 5 and 6 are not labeled in Figure 1.**

     >> Done.

**In the section of “conflicting data in comparison to previous studies” line 9, the statement “… from another E-protein of the same subunit” is inaccurate as subunit refers to a single polypeptide chain.**

     >> The word "subunit" has been replaced with "complex".

 I would like to thank you once again for the positive comments.

 best wishes,

 Sandeep

# Peer review: Referee response for version 1

**DOI:** 10.5256/f1000research.9528.r15004

**Published:** 2016-07-28

**Authors:** Raju Nagarajan

**Affiliations:** 1 Vanderbilt Vaccine Center, Vanderbilt Medical Center, Nashville, TN, USA

The author performed an essential structural analysis of ZIKV and DENV envelope proteins and reported the identification of unreported epitope residues with conservation details. The quality of writing and analysis is acceptable with some minor corrections.

1. The text is not much visible in Figure 6 and has to be revised.
2. In the caption of figure 5, it should be "192-195" instead of "192-95".
3. Title should be shortened.
4. The first sentence in the "Analysis of secondary structures" has to be revised to ensure that the analysis only performed for envelope proteins.

I have read this submission. I believe that I have an appropriate level of expertise to confirm that it is of an acceptable scientific standard.

# Response

**Published:** 2016-09-08

**Authors:** Sandeep Chakraborty

**Affiliations:** Tata Institute of Fundamental Research, India

Dear Dr Nagarajan,

 I would like to thank you for taking the time to review this paper, and providing constructive criticism on the overall manuscript. I have revised the manuscript based on the comment of all three reviewers.

 Please find my responses below.

**The author performed an essential structural analysis of ZIKV and DENV envelope proteins and reported the identification of unreported epitope residues with conservation details. The quality of writing and analysis is acceptable with some minor corrections.**

****

**The text is not much visible in Figure 6 and has to be revised.**

     >> This figure is high resolution, and text is visible clearly. I will request the typesetting to increase the size of this figure in the pdf.

**In the caption of figure 5, it should be "192-195" instead of "192-95".**

     >> Done.

**Title should be shortened.**

     >> Done.

**The first sentence in the "Analysis of secondary structures" has to be revised to ensure that the analysis only performed for envelope proteins.**

     >> Done.

 I would like to thank you once again for the positive comments.

 best wishes,

 Sandeep

# Peer review: Referee response for version 1

**DOI:** 10.5256/f1000research.9528.r14191

**Published:** 2016-06-15

**Authors:** Shamala Devi Sekaran, Keivan Zandi

**Affiliations:** 1 Department of Medical Microbiology, Faculty of Medicine, University of Malaya, Kuala Lumpur, Malaysia; 2 Department Of Medical Microbiology, Faculty of Medicine, University Malaya, Kuala Lumpur, Malaysia

1. The title needs to be shortened and revised as the should reflect the nature of the work which is all computational
2. Some other minor comments
  a. vaccine is only registered to date in 3 countries and not used by endemic regions as stated in line 4 of the abstract - please rewrite this.
  b. The last sentence has to be revised as I am not sure this addresses targets for drugs
  c. The last paragraph of introduction - was quantitative analysis of Den-2 E done with MEPP?
  d. Please check citation of APBS (version 1.4) which is given as Ref 52
  e. In the analysis of secondary structures - first sentence needs to revise as this was only done for the E protein and not all proteins

We have read this submission. We believe that we have an appropriate level of expertise to confirm that it is of an acceptable scientific standard.

# Response

**Published:** 2016-09-08

**Authors:** Sandeep Chakraborty

**Affiliations:** Tata Institute of Fundamental Research, India

Dear Dr Sekaran and Dr Zandi,

 I would like to thank you for taking the time to review this paper, and providing constructive criticism on the overall manuscript. I have revised the manuscript based on the comment of all three reviewers.

 Please find my responses below.

**The title needs to be shortened and revised as the should reflect the nature of the work which is all computational**

     >> Done.

**Some other minor comments**

****

**a. vaccine is only registered to date in 3 countries and not used by endemic regions as stated in line 4 of the abstract - please rewrite this.**

             >> Done

**b. The last sentence has to be revised as I am not sure this addresses targets for drugs**

             >> Revised. I have made the changes in the main manuscript too.

**c. The last paragraph of introduction - was quantitative analysis of Den-2 E done with MEPP?**

             >> Yes.

**d. Please check citation of APBS (version 1.4) which is given as Ref 52**

             >> Ref 52 was intended to direct the reader to the parameters used. I have cited APBS before this reference to remove the confusion.

**e. In the analysis of secondary structures - first sentence needs to revise as this was only done for the E protein and not all proteins**

             >> Fixed.

 I would like to thank you once again for the positive comments.

 best wishes,

 Sandeep
